# Relation Between Educational Attainment and Cerebral Small Vessel Disease: the Framingham Heart Study

**DOI:** 10.1002/alz.088476

**Published:** 2025-01-09

**Authors:** Matthew C Kang, Adlin A Pinheiro, Hugo J. Aparicio, Alexa S Beiser, Matthew P. Pase, Riya Manchanda, Charles Decarli, Sudha Seshadri, Serkalem Demissie, Jose Rafael Romero

**Affiliations:** ^1^ Boston University School of Medicine, Boston, MA USA; ^2^ Framingham Heart Study, Framingham, MA USA; ^3^ The Framingham Heart Study, Framingham, MA USA; ^4^ Boston University, Boston, MA USA; ^5^ Boston University Chobanian & Avedisian School of Medicine, Boston, MA USA; ^6^ Boston University School of Public Health, Boston, MA USA; ^7^ Turner Institute for Brain and Mental Health & School of Psychological Sciences, Monash University, Clayton, VIC Australia; ^8^ University of California, Davis School of Medicine, Sacramento, CA USA; ^9^ Department of Neurology, Boston University School of Medicine, Boston, MA USA

## Abstract

**Background:**

Socioeconomic factors have an impact on long‐term neurovascular health. Education attainment (EA) is a socioeconomic factor which may be related to vascular risk factors and brain health. We investigated the relation of EA and cerebral small vessel disease (CSVD) in community dwelling participants free of stroke and dementia.

**Method:**

We evaluated 3367 Framingham Heart Study Original and Offspring Cohort participants, categorizing EA into four levels (less than high school, high school graduate, some college, or more). CSVD was assessed via MRI, considering covert infarcts, cerebral microbleeds, cortical superficial siderosis, high perivascular space burden, and extensive white matter hyperintensities, with 1 point assigned for each, to categorize CSVD scores as 0, 1, or 2+. We related education level to the CSVD score using logistic regression analysis with a generalized logits model adjusted for age, sex, and cohort.

**Result:**

Participants without high school degrees had higher CSVD scores (23% had CSVD score 2+ vs 7% in college graduates) and a higher proportion of hypertension and diabetes. There were slightly higher odds of CSVD scores 2+ in those without high school degrees (OR 1.30, 95% CI 0.62 ‐ 2.73 vs. OR 0.81, 95% CI 0.59‐1.12 in participants with some college or more), but not statistically significant after multivariable adjusted analyses. There was a non‐statistically significant suggestion of higher odds of CSVD 2+ among participants with low EA with APOE‐𝜺4 alleles (OR 2.88, 95% CI 0.57‐14.45).

**Conclusion:**

Our findings suggest that lower EA may be linked to CSVD burden, but analyses were limited by a small sample of participants with highest CSVD burden and low EA. Further studies are needed to characterize the role of education, a modifiable risk factor, on vascular brain injury.

